# Suppression of a single BAHD gene in *Setaria viridis* causes large, stable decreases in cell wall feruloylation and increases biomass digestibility

**DOI:** 10.1111/nph.14970

**Published:** 2018-01-08

**Authors:** Wagner R. de Souza, Polyana K. Martins, Jackie Freeman, Till K. Pellny, Louise V. Michaelson, Bruno L. Sampaio, Felipe Vinecky, Ana P. Ribeiro, Barbara A. D. B. da Cunha, Adilson K. Kobayashi, Patricia A. de Oliveira, Raquel B. Campanha, Thályta F. Pacheco, Danielly C. I. Martarello, Rogério Marchiosi, Osvaldo Ferrarese‐Filho, Wanderley D. dos Santos, Robson Tramontina, Fabio M. Squina, Danilo C. Centeno, Marília Gaspar, Marcia R. Braga, Marco A. S. Tiné, John Ralph, Rowan A. C. Mitchell, Hugo B. C. Molinari

**Affiliations:** ^1^ Embrapa Agroenergy Brasília DF 70770901 Brazil; ^2^ Plant Sciences Rothamsted Research Harpenden, Hertfordshire AL5 2JQ UK; ^3^ Department of Biochemistry State University of Maringá Maringá, Paraná 87020‐900 Brazil; ^4^ Brazilian Bioethanol Science and Technology Laboratory Brazilian Center for Research in Energy and Materials Campinas, Sao Paulo 13083‐100 Brazil; ^5^ Programa de Processos Tecnológicos e Ambientais Universidade de Sorocaba (UNISO) Sorocaba 18060‐000 Brazil; ^6^ Centre of Natural Sciences and Humanities Federal University of ABC São Bernardo do Campo SP 09606‐045 Brazil; ^7^ Department of Plant Physiology and Biochemistry Institute of Botany Sao Paulo 04301‐012, 04301‐902 Brazil; ^8^ Department of Biochemistry University of Wisconsin Madison WI 537 USA; ^9^ Department of Energy's Great Lakes Bioenergy Research Center Wisconsin Energy Institute University of Wisconsin Madison WI 537 USA

**Keywords:** cell wall acylation, ferulic acid, grass evolution, hydroxycinnamates, lignocellulosic feedstock

## Abstract

- Feruloylation of arabinoxylan (AX) in grass cell walls is a key determinant of recalcitrance to enzyme attack, making it a target for improvement of grass crops, and of interest in grass evolution. Definitive evidence on the genes responsible is lacking so we studied a candidate gene that we identified within the BAHD acyl‐CoA transferase family.
- We used RNA interference (RNAi) silencing of orthologs in the model grasses *Setaria viridis* (*SvBAHD01*) and *Brachypodium distachyon* (*BdBAHD01*) and determined effects on AX feruloylation.
- Silencing of *SvBAHD01* in *Setaria* resulted in a *c*. 60% decrease in AX feruloylation in stems consistently across four generations. Silencing of *BdBAHD01* in *Brachypodium* stems decreased feruloylation much less, possibly due to higher expression of functionally redundant genes. *Setaria SvBAHD01* RNAi plants showed: no decrease in total lignin, approximately doubled arabinose acylated by *p*‐coumarate, changes in two‐dimensional NMR spectra of unfractionated cell walls consistent with biochemical estimates, no effect on total biomass production and an increase in biomass saccharification efficiency of 40–60%.
- We provide the first strong evidence for a key role of the *BAHD01* gene in AX feruloylation and demonstrate that it is a promising target for improvement of grass crops for biofuel, biorefining and animal nutrition applications.

## Abstract 2

See also the Commentary on this article by Gómez & McQueen‐Mason, **218**: 5–7.

## Introduction

Billions of tonnes of biomass, composed principally of secondary cell walls, are produced worldwide by grass crops annually either as the primary product for animal feed or as residues from food crops. Digestibility of this biomass – the ease with which sugar can be released from the cell‐wall polysaccharides – is a key economic target, both for the production of liquid biofuel and for efficiency of digestion by ruminant animals. A major distinguishing feature of grass cell walls and those of other commelinid monocots is the prevalence of two hydroxycinnamates, *p*‐coumarate (*p*CA) and ferulate (FA) (Harris & Trethewey, 2010). FA, in particular, is heavily involved in grass cell‐wall cross‐linking reactions. The FA acylates arabinofuranosyl units that are 1→3‐linked to the xylan backbone in arabinoxylan (AX) or glucuronoarabinoxylan (GAX). Ester‐linked FA oxidatively couples in a similar manner to that of lignin monomers (Ralph *et al*., 1992, 1995), forming cross‐links with other (G)AX chains or with lignin (Ishii, 1997; Ralph *et al*., 1998, 2004; Ralph, 2010). These cross‐links inhibit digestion by preventing enzyme access and by tightly binding the polysaccharide substrate to nondigestible lignin. Decreasing FA content and thereby FA‐mediated cross‐linking of grass biomass has therefore long been considered a promising target for increasing digestibility (de Oliveira *et al*., 2015) and this is supported by: *in vitro* studies showing inhibition by FA of polysaccharide saccharification to sugars (Grabber *et al*., 1998a,b); natural variation in FA content being inversely correlated with digestibility (Lam *et al*., 2003; Casler & Jung, 2006); increasing biomass digestibility by heterologous expression of feruloyl esterase (Buanafina *et al*., 2008); and screens for increased digestibility in mutant populations that frequently identify low ferulate lines (e.g. Hirano *et al*., 2017).

**Figure 1:**
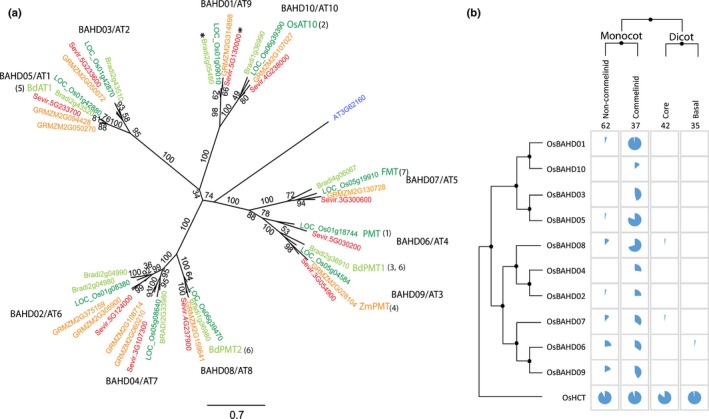
(a) Phylogenetic tree of candidate clade of BAHD genes (Mitchell *et al*., 2007) showing BAHD names for each branch from Molinari *et al*. (2013) and alternative AT names from Bartley *et al*. (2013). All genes from Arabidopsis, rice, *Brachypodium*, maize and *Setaria* in sub‐clade A are shown. Support for the topology is shown as a percentage of bootstrap runs. Named genes have evidence on function from: 1, Withers *et al*. (2012); 2, Bartley *et al*. (2013); 3, Petrik *et al*. (2014); 4, Marita *et al*. (2014); 5, Buanafina *et al*. (2016); 6, Sibout *et al*. (2016); 7, Karlen *et al*. (2016). Asterisks mark the genes we studied here. (b) Distribution of orthologs present in the 1KP project (Matasci *et al*., 2014) to the candidate rice genes and to related OsHCT genes. Proportions of species (out of the total number shown at the top of the grid) that have orthologs are shown as blue pie chart slices.

Candidate genes involved in feruloylation of AX were first identified by differential expression between grasses and dicots (Mitchell *et al*., 2007) as residing within a clade of genes in the BAHD acyl‐coenzyme A (CoA) transferase superfamily (there named ‘PF02458 family’ after the characteristic PFAM domain). The most likely candidate within this clade for involvement in AX feruloylation based on absolute expression level and coexpression was identified as the rice gene LOC_Os01g09010, which we call here OsBAHD01. There are orthologs for this gene in all sequenced commelinid monocots; in *Brachypodium distachyon* (Brachypodium), *Setaria viridis* (Setaria) and maize there is a one‐to‐one ortholog (Fig. 1a). Suppression of OsBAHD01 by RNA interference (RNAi) in rice was correlated with decreased cell‐wall FA (Piston *et al*., 2010); however, the FA decrease was variable between tissues and generations (largest decrease was 27% in stems of one line) and the construct was designed to suppress four other closely related genes as well as BAHD01. There is now strong evidence that one of these (AT10) is specifically responsible for the acylation of AX by *p*CA rather than FA (Bartley *et al*., 2013). *p*CA is a hydroxycinnamate, like FA, but crucially does not readily oxidatively couple *in vivo* and therefore does not participate extensively in cross‐links, although it may facilitate lignin polymerization (Ralph, 2010). Others genes in the clade may also be responsible for AX feruloylation; RNAi suppression and overexpression of BdBAHD05 (Fig. 1A; BdAT1 in the nomenclature of Bartley *et al*. (2013)) induced decreases and increases, respectively, in FA in transgenic *Brachypodium* lines (Buanafina *et al*., 2016), although the effects were relatively small (lines with the largest effects showed a *c*. 25% decrease for RNAi, *c*. 15% increase for overexpression). Effects on cell‐wall FA or *p*CA due to manipulation of gene expression could be indirect, for example, due to perturbation of metabolite concentrations; this interpretation is made more plausible for the Piston *et al*. (2010) and Buanafina *et al*. (2016) studies by the modest size of effects on FA. Alternatively, they may be directly responsible for AX feruloylation, but compensatory mechanisms may operate or there may be gene redundancy within the clade. Several of the genes are now known to encode enzymes that acylate monolignols rather than AX by FA or *p*CA (Withers *et al*., 2012; Petrik *et al*., 2014; Karlen *et al*., 2016; Sibout *et al*., 2016), leaving BAHD05 as putatively functionally redundant with BAHD01 and three others (BAHD02, BAHD03, BAHD04; Fig. 1a) with no functional indications.

*S. viridis* is an emerging monocot plant model for molecular and genetic studies. It is a short, fast‐growing, C_4_ plant and its genome sequence is fully available (Bennetzen *et al*., 2012). It is the same sub‐family Panicoideae as sorghum, maize and sugarcane. In addition, *S. viridis* is amenable to genetic transformation through *Agrobacterium tumefaciens* (Martins *et al*., 2015). *Brachypodium* is a model C_3_ grass species in the same BOP clade of Poaceae as rice, wheat and *Lolium* (Vogel *et al*., 2010).

Here we show the effects of suppressing SvBAHD01 and BdBAHD01 expression in *Setaria* and *Brachypodium*, respectively. Although the effects on cell‐wall FA in *Brachypodium* are of similar magnitude to those reported previously for effects of BAHD suppression, those in *Setaria* are much larger and more consistent. We investigate possible reasons for this by examining the RNA‐seq in transgenics of the two species. We also characterize the effects on cell walls, growth and digestibility of biomass in the *Setaria* transgenics and discuss the likely role of BAHD01 genes.

## Materials and Methods

### Phylogenetic analysis

We downloaded protein sequences from Phytozome 12 (Goodstein *et al*., 2012) for the rice and *Brachypodium* BAHD candidate sequences identified (Mitchell *et al*., 2007; Molinari *et al*., 2013) and their orthologs in maize and *Setaria*. The BdBAHD04 gene model is incorrect in *Brachypodium* v3 as shown by strand‐specific RNA‐seq so we replaced it with the v1 model. We performed alignment then optimization of topology, parameters and branch length followed by bootstrapping as previously described (Pellny *et al*., 2012) but using PhyML3.0 (Guindon *et al*., 2010). To identify orthologs in the 1,000 Plants (1KP) database of plant transcriptomes (Wickett *et al*., 2014), we identified all hits with *E* < 10^−5^ using nucleotide Blast (Blastn) (Matasci *et al*., 2014) with rice candidate genes as queries; these were downloaded and assigned as ortholog of top rice hit if the bit score was > 100 using translated nucleotide searches against the rice proteome with Tera‐Blastp on the DeCypher ^®^ platform.

### Plasmid construct and generation of transgenic plants

For silencing of BAHD01 in both *Setaria* and *Brachypodium*, we selected a 254 bp sequence with identical matches to both SvBAHD01 and BdBAHD01 (Supporting Information Fig. S1A) and no off‐target identical matches of > 16 bp. Inverted repeats of this 254 bp flanking the maize Adh2 intron were synthesized by Genscript (Piscataway, NJ, USA), and subcloned into the transformation vector A224p6i‐U‐Gusi, using standard cloning techniques, giving rise to the plasmid plTY73. In plTY73, the BAHD01 RNAi cassette is under control of the maize ubiquitin promoter. We transformed *Brachypodium* inbred line Bd21 and *S. viridis* accession A10.1 following published protocols (Vogel & Hill, 2008) and (Martins *et al*., 2015), respectively.

### RNA sequencing and differential expression analysis

For *Setaria*, mRNA of stem tissue from three replicate plants was obtained using an NEBNext^®^ RNA Library Prep Set Kit for Illumina^®^. Libraries were made using an NEB Next^®^ Ultra RNA Library Prep Kit and sequenced on a HiSeq4000 using TruSeq SBS v3 kit (Illumina) by GenOne Biotechnologies. For *Brachypodium*, total RNA was isolated from stems of a minimum of three replicate plants using the protocol of Chang *et al*. (1993). Libraries were made using a Ion Total RNA‐Seq Kit v2, templates were prepared using the Ion PITM Template OT2 200 Kit V2 and were sequenced using the Ion PITM Sequencing 200 Kit v2 with an Ion PITM Chip Kit v2 on an Ion ProtonTM System. All sequencing equipment and reagents were from Thermo Fisher Scientific and were used following the manufacturer's instructions. For both *Setaria* and *Brachypodium*, reads were mapped to reference transcriptomes from Phytozome 11.0 using the BWA‐MEM algorithm (Li & Durbin, 2010) with default parameters for *Setaria* and accepting forward reads only for the strand‐specific reads generated by Ion Proton sequencing for *Brachypodium*. Expression measures FPKM (fragment per kilobase per million mapped reads) and CPM (counts per million) were generated by eXpress (Roberts & Pachter, 2013); global analysis to identify all differentially expressed genes was performed using the edgeR package in R (Robinson *et al*., 2010). All reads and protocols have been deposited in the ArrayExpress public database under accession E‐MTAB‐5648 for *Setaria* and E‐MTAB‐5649 for *Brachypodium*.

### Quantification of cell‐wall‐bound hydroxycinnamate content

**Figure 2:**
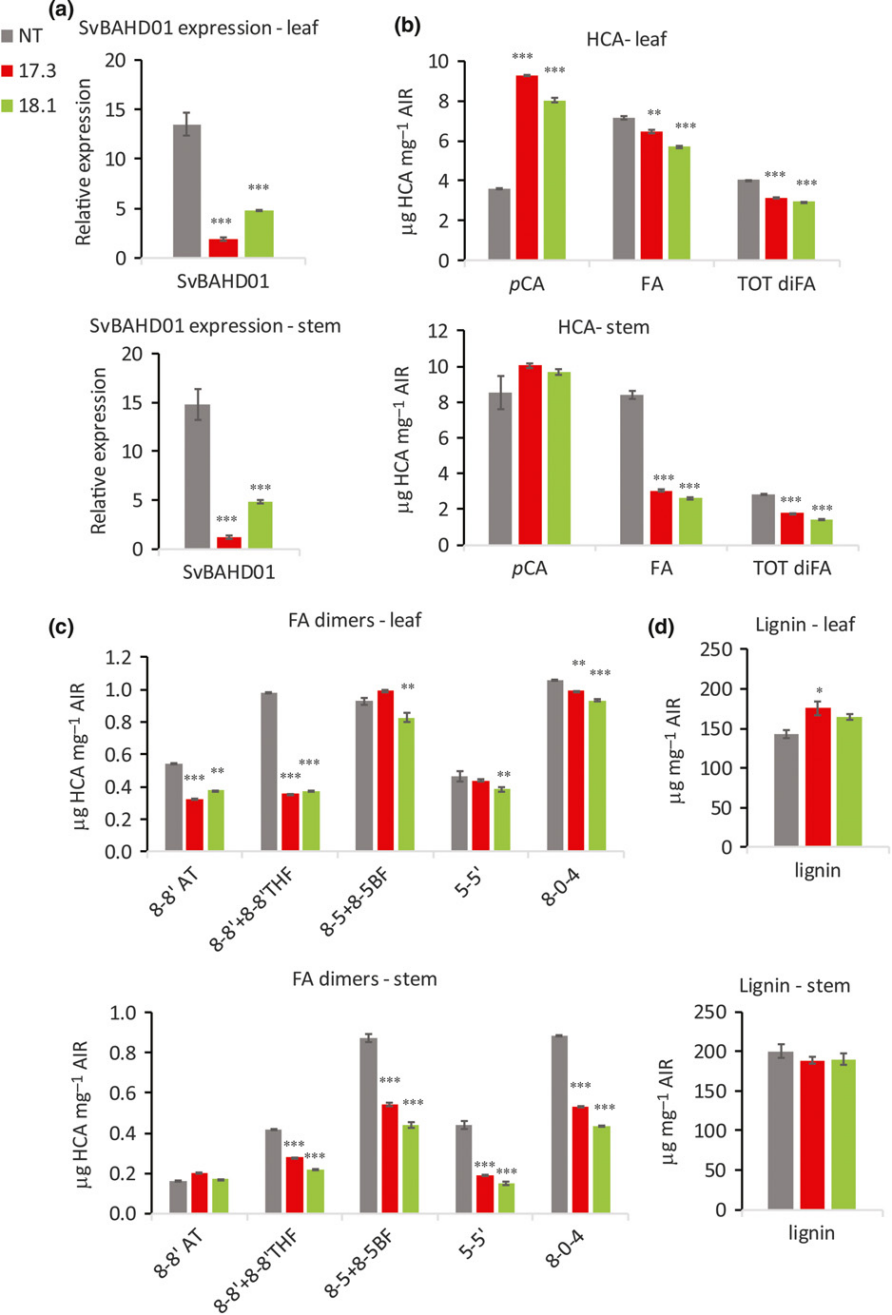
SvBAHD01 gene expression (a) and ester‐linked HCA and lignin content (b–d) of cell walls in leaves and stems of *Setaria* control (NT) and T_3_ plants from the 17.3 and 18.1 RNAi‐silenced lines (*n *=* *3; error bars ± SEM; significance of difference of transgenic from control indicated if difference in means > least significant difference from ANOVA at: *, *P *<* *0.05; **, *P *<* *0.01; ***, *P *<* *0.001).

Cell‐wall‐bound hydroxycinnamate (HCA) content was determined essentially as described (Freeman *et al*., 2017) in labs at Embrapa Agroenergy (Supporting Information Table S1; Fig. S1C) and Rothamsted (Figs 2, S1D; Table S2) and with some variations in protocol as described in Methods S1.

### Determination of HCA conjugates released by mild acidolysis

Alcohol‐insoluble residue (AIR) was prepared using extractions as described for cell‐wall‐bound HCA and then treated with 1.2 ml 50 mM trifluoroacetic acid (TFA) for 4 h at 99°C with agitation at 750 rpm. After centrifugation for 10 min at 16 000 ***g***, two 500 μl aliquots of supernatant were freeze‐dried. The pellet was washed twice with water and freeze‐dried. Released HCA conjugates from one 500 μl aliquot of supernatant were dissolved in 250 μl 50% methanol : 0.1% formic acid and 10 μl separated as for cell‐wall‐bound HCA except using a binary gradient with methanol (solvent A) and 0.1% formic acid (solvent B) with the following gradient: isocratic 100% B, 0–1 min; linear 100% to 0% B, 1–21 min; isocratic 0% B, 21–23 min; linear 0% to 100% B, 23–28 min with a flow rate of 1 ml min^−1^. (We found this column provided much improved resolution over that used by Bartley *et al*. (2013)). For mass spectrometry analysis samples were diluted 1 : 8 and 10 μl was analyzed on a 4000 QTRAP LC‐MS/MS system (SCIEX, Framingham, MA, USA) after HPLC using an Agilent 1200 fitted with a 100 μl sample loop. The probe was vertically positioned 11 mm from the orifice and charged at −4500 V. Temperature was held at 750°C, GS1 was set at 20 psi, GS2 at 20 psi, curtain gas at 20 psi and the interface heater was engaged. Multiple reaction monitoring (MRM) transitions were derived from standards, previously published data (Quemener & Ralet, 2004; Bartley *et al*., 2013) and experimentally. Declustering potential, entrance potential, collision energy and collision cell exit potential were set on an analyte‐dependent basis (Table S3). Data were collected with Analyst (SCIEX) software and integrated using the Intelliquant algorithm. Peaks for individual analytes were assigned based on their MRM transitions.

#### Quantitation of Ara‐FA and Ara‐*p*CA

**Figure 3:**
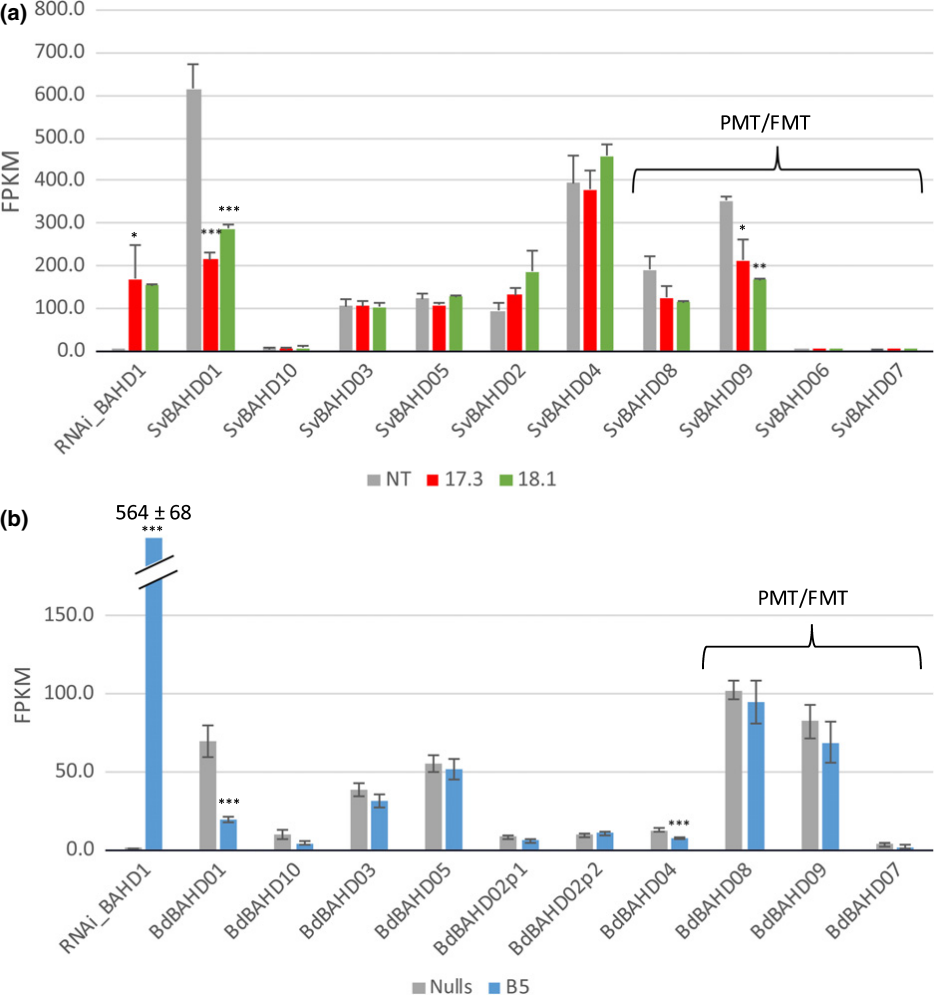
RNA‐seq analysis of BAHD gene expression in (a) *Setaria* SvBAHD01 RNAi lines and (b) *Brachypodium* BdBAHD01 RNAi lines. Genes associated with monolignol acylation (PMT and FMT) are indicated. Transcript abundance is measured as fragment per kilobase per million mapped reads (FPKM;* n *=* *3; error bars ± SEM; significance of difference of transgenic from control indicated if difference in means > least significant difference from ANOVA at: *, *P *<* *0.05; **, *P *<* *0.01; ***, *P *<* *0.001).

Samples from mild acidolysis were run on a Shimadzu Prominence HPLC device with a photo‐diode array detector using the same column and protocol as for LC‐MS. Areas for peaks (absorbance at 280 nm) at retention times corresponding to Ara‐FA and Ara‐*p*CA ions show the same relative effects as ion counts across samples (Fig. S2). Peak area relative to internal standard peak area was used to quantify the peaks, using calibrations of corresponding free HCA with pure standards. Values were multiplied by a correction factor for the difference in absorbance of Ara‐HCA from free HCA, derived as follows. Fractions for Ara‐FA and Ara‐*p*CA peaks were collected and split into two equal samples, one of which was saponified, the other untreated. They were then re‐run on the HPLC device and correction factors were calculated as (peak area free HCA)/(peak area Ara‐HCA) giving values of 0.92 and 0.62 for *p*CA and FA, respectively. This quantitation of Ara‐HCAs was conducted in the Rothamsted lab to give the data shown in Fig. 3. The same procedure was used in Embrapa Agroenergy to give relative amounts in Fig. S3, but using a Waters ACQUITY UPLC device (Milford, MA, USA). To determine total ester‐linked HCA following mild acidolysis (Table S4), aliquots of supernatant and the pellet were saponified, dried under vacuum, resuspended in 250 μl 50% methanol : 0.1% formic acid, and HCA content was quantified as in Freeman *et al*. (2017) but using the HPLC method above used to separate HCA conjugates.

### Cell wall characterization by solution‐state two‐dimensional NMR

We characterized the cell walls of *Setaria* samples without fractionation using solution‐state two‐dimensional (2D) NMR following the procedure described by Kim & Ralph (2010); full details are given in Methods S1.

### Enzymatic saccharification assay

Samples of leaf and stem tissues of the lines Ev.17.3, Ev.18.1 and nontransfromed (NT) at the reproductive stage were ground in a ball mill for 30 s and then subjected to enzymatic saccharification with a commercially available enzyme preparation CellicCtec2 (Novozymes, Lyngby, Denmark) at 10 filter paper cellulase units g^−1^ biomass. For biomass pre‐treatment, 0.25% H_2_SO_4_ was added and samples were incubated at 120°C and 3.5 bar for 30 min. The enzymatic hydrolysis (EH) experiments were performed using 2 ml Eppendorf tubes with 2% (w/v) biomass in 100 mM phosphate buffer, pH 5.0, and 0.1% sodium azide as an antimicrobial agent. The reaction was incubated in a Thermomixer microplate incubator (Eppendorf, Germany) operated at 50°C and agitation speed of 1000 rpm. Samples were withdrawn after 6 h, and the hydrolysis was stopped by heating the samples at 80°C for 5 min, followed by centrifugation at 10 000 ***g*** for 15 min. EH was measured by quantification of the glucose released according to the glucose oxidase assay (Kit MAK097; Sigma‐Aldrich, St Louis, MO, USA) according to the manufacturer's instructions .

### Other methods

Procedures based on published methods for determination of gene expression (Martins *et al*., 2016), cell‐wall monosaccharides (Sluiter *et al*., 2012), lignin (Moreira‐Vilar *et al*., 2014), growth conditions, biomass and stem microscopy are described in detail in Methods S1.

## Results

### Identification of candidate BAHD gene for cell‐wall feruloylation and generation of silencing lines in *Setaria* and *Brachypodium*

We analyzed the phylogeny of *BAHD* genes in the candidate clade (sometimes called ‘Mitchell Clade’ (Bartley *et al*., 2013)) for maize, *Brachypodium*,* Setaria*, rice and Arabidopsis (Fig. 1a). (Sub‐clade B genes that show low expression, much less conservation between species and no co‐expression with cell‐wall genes (Molinari *et al*., 2013) are omitted.) Of the genes without direct functional evidence, BAHD01 is a good candidate for cell‐wall feruloylation (Mitchell *et al*., 2007; Bartley *et al*., 2013; Molinari *et al*., 2013). Orthologs of OsBAHD01 are present in nearly all commelinid monocots, but are almost completely absent from transcriptomes of other angiosperms present in the 1KP database (Matasci *et al*., 2014; Fig. 1b), consistent with the taxonomic distribution of (G)AX feruloylation (Harris & Trethewey, 2010). We therefore selected Sevir.5G130000 (SvBAHD01) and its ortholog Bradi2g05480 (BdBAHD01) as targets for suppression. We created a construct with an RNAi hairpin designed to suppress both SvBAHD01 and BdBAHD01 under control of a constitutive maize ubiquitin promoter (Fig. S1A). We transformed *Setaria* with this construct, obtaining seven independent lines silencing SvBAHD01 by 61–99% in leaves that showed decreases in FA content of 39–60% (Table S1). We performed segregation analysis through two generations (T_2_ plants), obtaining four independent stable homozygous lines compatible with a single locus for the Sv*BAHD01* RNAi transgene (Table S1). We observed decreased FA contents in the cell walls of leaf and stem tissues in these transgenic lines (Fig. S1C). We selected two of the best performing lines, 17.3 and 18.1, for further detailed analysis. We also generated six independent homozygous BdBAHD01 RNAi lines in *Brachypodium*; these showed only small effects on FA and *p*CA (Table S2) and we selected two of these for further analysis.

### Cell‐wall HCA contents of SvBAHD01 RNAi *Setaria* plants and BdBAHD01 *Brachypodium* RNAi plants

To test the stability of *BAHD01* gene silencing and phenotype inheritance of altered HCA composition in the cell walls of *Setaria*, we analyzed T_3_ generation transgenic plants from lines 17.3 and 18.1. Silencing levels were maintained in these plants; Sv*BAHD01* expression compared with nontransfromed (NT) plants from lines 17.3 and 18.1 were 82% and 64% lower in leaves and 90% and 70% lower in stems, respectively (Fig. 2a). We observed a similar reduction in FA contents in the cell walls of stem as seen in the T_0_ and T_2_ generations, corresponding to > 60% decrease compared to NT plants, but the decrease in FA in leaves was smaller than in T_2_ (Fig. 2b). We found that ester‐linked *p*CA was more than double in the cell walls of leaves for lines 17.3 and 18.1 compared to control, whereas in the cell walls of stems we found only a small increase (Fig. 2b). We also quantified the amounts of five different forms of dehydrodiferulates (diFAs); in leaves, the 8–8′‐diFA coupled forms (8‐8′, 8‐8′ THF and 8‐8′ AT) were substantially decreased, with other diFAs unaffected (Fig. 2c). By contrast, all diFAs except the aryltetralin form of 8–8′‐diFA (8‐8′ AT) were decreased in stems (Fig. 2c). Dimerization expressed as sum of all diFAs over total FA was unaffected in leaves but highly significantly (*P *<* *0.001) increased in stems in both lines (NT: 25%, 17.3: 37%, 18.1: 35%). We found lignin content, as assessed by the acetyl bromide method, to be unchanged in stems of SvBAHD01 RNAi plants but with a modest increase in leaves (Fig. 2d).

We also analysed two *Brachypodium* BdBAHD01 RNAi lines for HCA content; these also showed small (10–20%), but significant, decreases in FA and diFA content of stem cell walls (Fig. S1E).

### Transcriptome analysis of transgenic SvBAHD01 and BdBAHD01 RNAi plants

We analyzed the RNA‐seq transcriptomes from stems of T_3_ SvBAHD01 and BdBAHD01 RNAi plants to diagnose the difference in magnitude of the effects of the transgenes in *Setaria* and *Brachypodium*, and also to test for off‐target and pleiotropic effects. We observed similar relative decreases in expression of BdBAHD01 in *Brachypodium* line B5 (78%) as of SvBAHD01 in *Setaria* lines 17.3 and 18.1 (65% and 53%) (Fig. 3). However, expression of SvBAHD01 was much greater than that of BdBAHD01 in control lines, both in absolute FPKM values and, importantly, relative to similar genes (Fig. 3), some of which may be functionally redundant such as BAHD05 (Buanafina *et al*., 2016). Greater relative expression of redundant genes in *Brachypodium* could explain the much smaller effect of BdBAHD01 suppression compared to SvBAHD01 suppression. We found no evidence of a compensatory increase in expression of any BAHD genes in response to BAHD01 suppression. SvBAHD09, an ortholog of BdPMT1, was significantly down‐regulated (Fig. 3); because SvBAHD09 and the RNAi construct shared little identity (longest identical sequence is 12 bp), we interpret this as a pleiotropic effect of suppressing SvBAHD01. Many other genes were also significantly differentially regulated due to pleiotropic effects in both 17.3 and 18.1 (Notes S1), including upregulated genes associated with negative regulation of transcription and protein synthesis and downregulated genes associated with cytoskeleton and xylan synthesis and remodeling.

As the effects of BAHD01 suppression were much greater in *Setaria* than in *Brachypodium*, we focused on characterizing the SvBAHD01 RNAi lines in more detail.

### Characterization of xylan in SvBAHD01 RNAi plants

**Figure 4:**
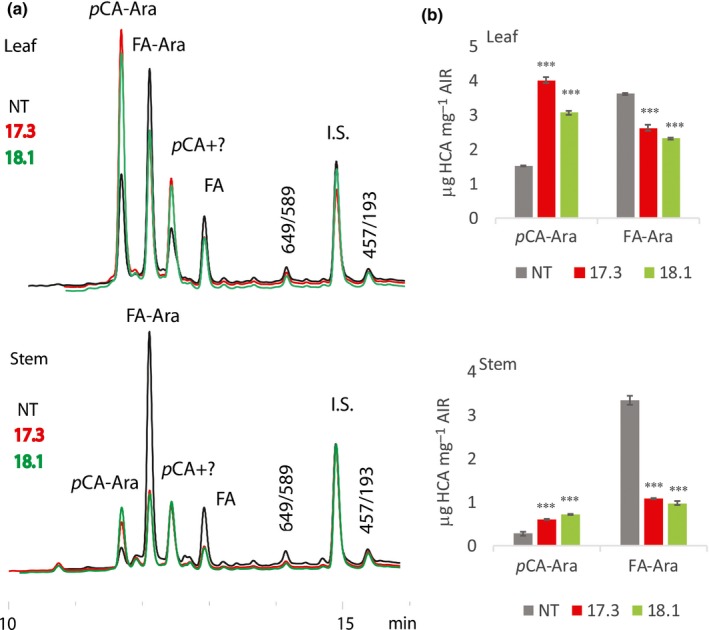
HCA conjugates in supernatant following mild acidolysis of *Setaria* alcohol‐insoluble residue (AIR). (a) Parts of representative HPLC chromatograms showing UV absorption. Major peaks for *p*‐coumarate (*p*CA)‐Ara and ferulate (FA)‐Ara were identified by LC‐MS (Supporting Information Fig. S2; Table S3). *p*CA co‐elutes with an unknown UV‐absorbing compound. Minor peaks are labeled according to their dominant parent/daughter ion *m*/*z* from LC‐MS; 649/589 and 457/193 are probably Ara‐diFA‐Ara and Xyl‐Ara‐FA, respectively. (b) Mean *p*CA‐Ara and FA‐Ara contents expressed as μg HCA equivalent per mg AIR estimated from similar chromatograms as shown in (a) (*n *=* *3; error bars ± SEM; significance of difference of transgenic from control indicated if difference in means > least significant difference from ANOVA at: ***, *P *<* *0.001).

Bound FA and diFA are ester‐linked to arabinofuranosyl units attached to GAX of grass cell walls (Scheller & Ulvskov, 2010); we found no consistent effect on Ara and Xyl content of AIR (predominantly derived from GAX) in SvBAHD01 RNAi plants (Table S5). We analysed Ara‐HCAs using mild acidolysis with TFA of AIR, which preferentially breaks glycosidic Ara‐(1→3)‐Xyl linkages in GAX, but can break other linkages releasing GAX oligosaccharides and free HCAs, and can chemically modify the Ara‐HCA (Saulnier *et al*., 1995; Bartley *et al*., 2013). We developed a novel method which identifies peaks from LC‐MS for Ara‐FA, Ara‐*p*CA and minor peaks with *m*/*z* of parent/daughter ions 649/589 and 457/193 (Table S3; Fig. 4a). These minor peaks are consistent with Ara‐diFA‐Ara and Xyl‐Ara‐FA (Table S3), being derived, respectively, from a xylan‐diFA‐xylan cross‐link and from the 2‐β‐Xyl*p*‐(5‐feruloyl)‐Ara*f* decoration of xylan that is common in grasses (Wende & Fry, 1997).

Using peak areas in ultraviolet absorbance spectra from this method, we found *c*. 30% and *c*. 70% decreases in Ara‐FA caused by SvBAHD01 silencing in leaves and stems, respectively (Fig. 4B). This Ara‐FA accounts for *c*. 40% of the FA monomer in all samples (Table S4), so relative effects are similar to those for FA (Fig. 2). (The remainder of the FA monomer in TFA‐treated samples is present in other forms; Tables S4, S5.) Ara‐*p*CA was increased after SvBAHD01 silencing to about double in both leaves and stems, although the absolute amount is low in stems (Fig. 4b).

To test the stability of the silencing, we repeated analyses of Ara‐HCAs in T_4_ generation plants (Fig. S3). We observed essentially the same effect of SvBAHD01 silencing on Ara‐FA as in T_3_: a *c*. 65% decrease in stems and *c*. 35% decrease in leaves. The increases in Ara‐*p*CA were more variable, ranging from 30% to 150% (Fig. S3).

Total ester‐linked *p*CA from cell walls comprises Ara‐*p*CA and lignin‐*p*CA, with lignin‐*p*CA being much more abundant in stems than in leaves. Lignin‐*p*CA is enriched in the pellet fraction following mild acidolysis; we found increases in *p*CA in this fraction in leaves of SvBAHD01 RNAi T_3_ and T_4_ plants (Table S4) to be of similar magnitude to the Ara‐*p*CA increases (Figs 4, S3), but in stems the increases were inconsistent and smaller. This suggests that lignin‐*p*CA was increased similarly to Ara‐*p*CA in leaves but not stems of SvBAHD01 RNAi plants.

### 2D‐NMR characterization of cell walls in SvBAHD01 RNAi plants

**Figure 5:**
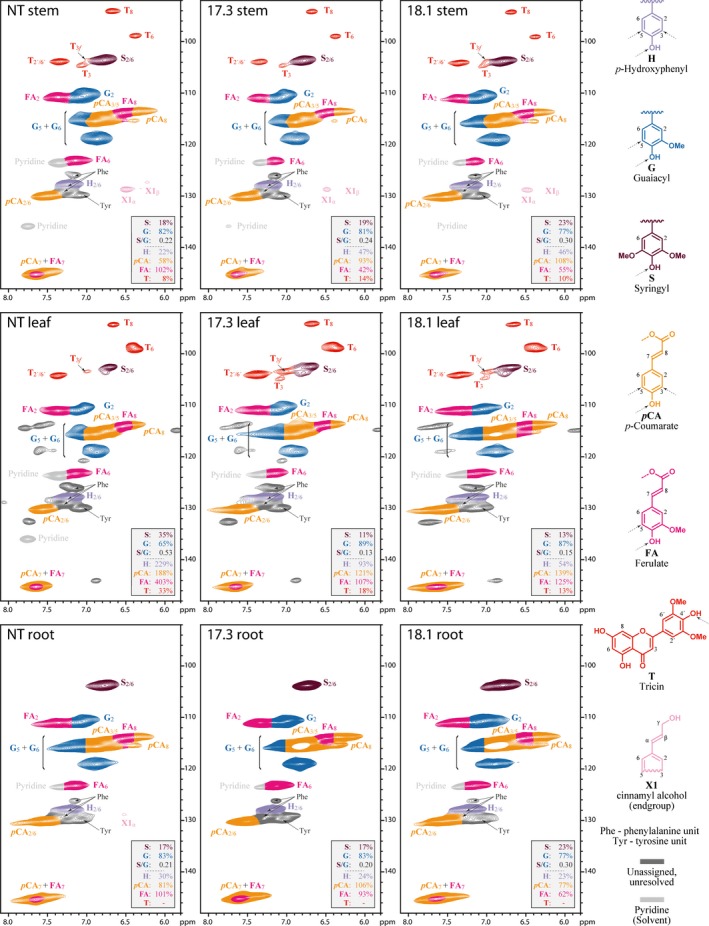
2D‐NMR heteronuclear single‐quantum coherence (HSQC) partial spectra of stem, leaf and root tissues from the WT control (NT) and the two transgenic lines (17.3 and 18.1) of *Setaria*. Color coding of the contours matches that of the assigned structures; where contour overlap occurs, the colorization is only approximate. The analytical data are from volume integrals of correlation peaks representing reasonably well‐resolved (except for **H**) C/H pairs in similar environments; thus, they are from **S**_2/6_, **G**_2_, **H**_2/6_, **FA**_2_, ***p*****CA**_2/6_ and **T**_2′/6′_, with obvious correction for those units that have two C/H pairs per unit. All relative integrals are on a G + S = 100% basis; H‐units are over‐quantified due to an overlapping peak from protein phenylalanine (Phe) units (Kim *et al*., 2017).

To gain information on the overall aromatic composition of the unfractionated cell walls in the SvBAHD01 silenced plants, we analyzed them using gel‐state 2D‐NMR (Kim & Ralph, 2010). We observed clear differences in the spectral fingerprints between control and SvBAHD01 RNAi plants in both leaves and stems, showing the expected decrease in FA peaks (Fig. 5). The magnitude of the decrease as estimated from the normalized integrals shown in Fig. 5 (*c*. 50%) is similar to that estimated from biochemistry (*c*. 60%; Fig. 2) for stems but much greater in leaves (*c*. 70% for 2D‐NMR compared to *c*. 10% in Fig. 2). The 2D‐NMR values are on a lignin basis and it is known that the integrals of small mobile components such as FA and *p*CA relative to those for relatively immobile internal lignin units are significantly over‐represented and variable in this methodology. The changes in FA : *p*CA ratios show better agreement between the 2D‐NMR and biochemical methods, −58% and −65% in leaves, and −71% and −73% in stems respectively. The smaller effect of SvBAHD01 silencing on this ratio in roots (−33%) compared to other tissues is also consistent with the smaller effect we saw on FA content of roots using the biochemical assay (Fig. S1C).

### Plant biomass, seed yield, saccharification and stem microscopy in SvBAHD01 RNAi plants

**Figure 6:**
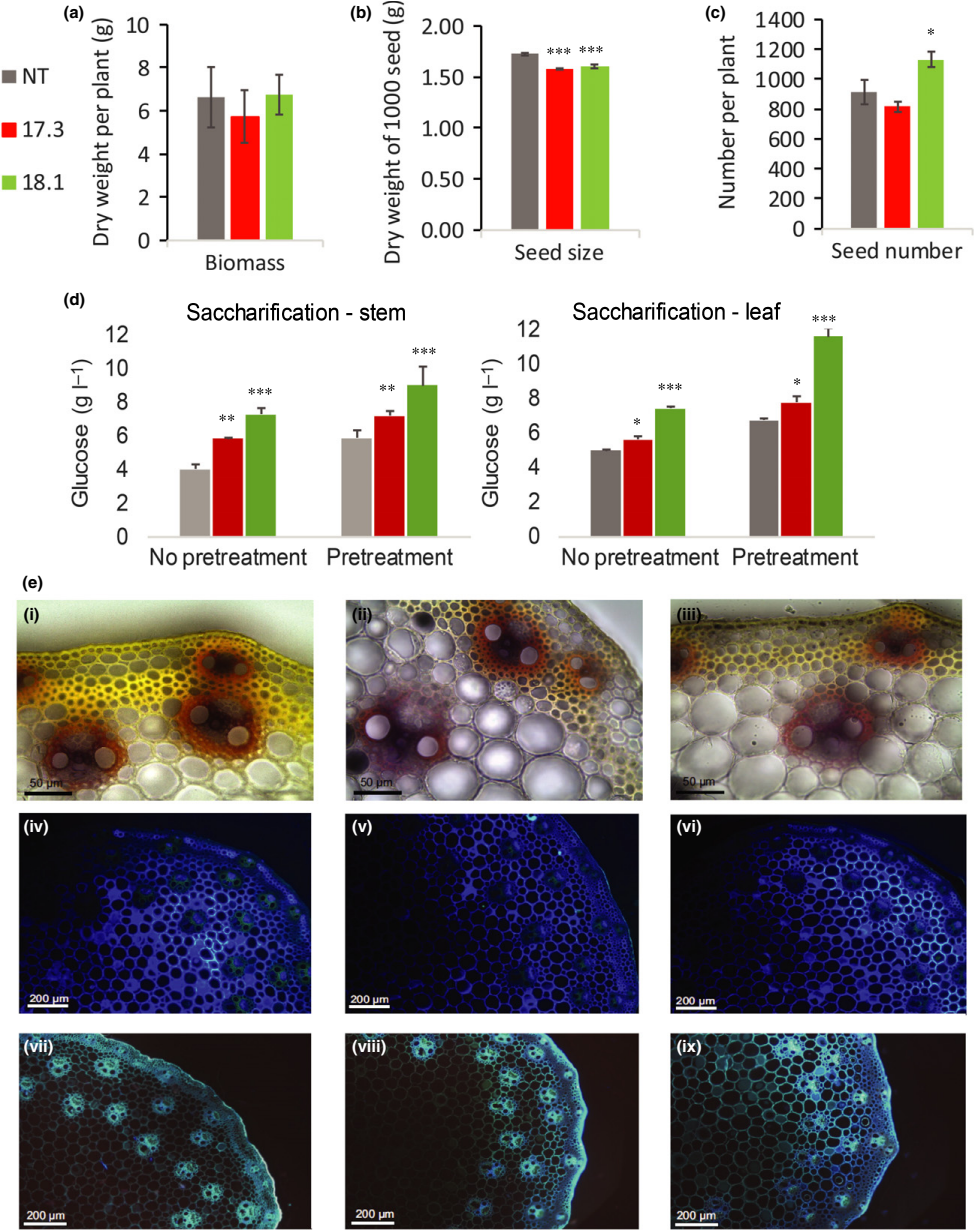
Biomass (a), seed size (b), seed number (c), saccharification (d) and stem morphology (e) of *Setaria SvBAHD01* RNAi plants. (a–d) Means ± SEM from 10 (a, b) or five (c, d) replicate plants, significance of difference of transgenic from control indicated if difference in means > least significant difference from ANOVA at: *, *P *<* *0.05; **, *P *<* *0.01; ***, *P *<* *0.001. (e) Representative stem sections from NT (i, iv, vii), 17.3 (ii, v, viii) and 18.1 plants (iii, vi, ix) stained with phloroglucinol (i–iii), auramine O (iv–vi) and showing autofluorsence (vii–ix).

We observed no significant changes in aerial biomass associated with the SvBAHD01 RNAi plants (Fig. 6a), but there was a significant small (8%) decrease in seed size (Fig. 6b) and an apparent increase in seed number in line 18.1 (Fig. 6c). We assessed ease of saccharification of biomass from transgenic plants pretreated or not with 0.25% H_2_SO_4_. Levels of glucose released by transgenic plants were significantly higher compared with NT plants, both for treated and for nontreated samples, in both tissues, indicating a more efficient saccharification of SvBAHD01 RNAi plant biomass (Fig. 6d). In cross‐sections of stems, the walls of sclerenchyma and parenchyma cells of SvBAHD01 RNAi plants were not as thick as those of control plants (Fig. 6e). We also observed a change in staining with phloroglucinol in interfascicular sclerenchyma, which were pale yellow in SvBAHD01 RNAi plants in contrast to bright yellow in controls (Fig. 6e i–iii), possibly related to the presence of benzaldehydes (Akin, 1990). Both staining with auramine O (Fig. 6e, iv–vi) and autofluorescence (Fig. 6e, vii–ix) of vascular bundle cells and interfascicular sclerenchyma were somewhat decreased, consistent with decreased cell wall phenolic content.

## Discussion

BAHD01 was identified as a candidate for involvement in the feruloylation of GAX 10 yr ago, along with other genes in the same clade (Mitchell *et al*., 2007). There was some early evidence for this role for BAHD01 from RNAi suppression of several genes together (BAHD01, 02, 04, 08 and 10) in rice in which decreases of FA of 10–30% were observed (Piston *et al*., 2010). However, this construct simultaneously suppressed expression of other genes including one since implicated in the addition of *p*CA, not FA, to GAX (BAHD10/AT10; Bartley *et al*., 2013) and an ortholog of BdPMT2 (BAHD08) putatively involved in addition of *p*CA to lignin (Sibout *et al*., 2016). We are not aware of any reports of knock‐out mutants for BAHD01, nor of *in vitro* activity assays for the encoded protein, both of which have been reported for the closely related lignin PMT genes (Withers *et al*., 2012; Marita *et al*., 2014; Petrik *et al*., 2014). Characterization of this gene has therefore lagged behind that of PMT despite the importance of feruloylation in determining properties of grass biomass.

Moderate changes, which vary between generations (Piston *et al*., 2010; Buanafina *et al*., 2016) in cell wall FA due to suppression and overexpression of BAHD genes, can be interpreted as secondary effects; however, the large, consistent effects that we observed from suppressing SvBAHD01 on FA content in *Setaria* generations T_0_, T_2_, T_3_ and T_4_ (Table S1; Figs 2, 4, S1C, S3) make this interpretation less plausible. Our results on the effect on cell‐wall FA of suppressing BdBAHD01 in *Brachypodium* were more like these previous reports in magnitude (Fig. S1E). Our transcriptome analysis offers one possible explanation for the greater magnitude of the effect in *Setaria* than in *Brachypodium*; SvBAHD01 is more highly expressed than BdBAHD01 relative to other candidate BAHD genes (Fig. 3). In particular, BdBAHD05 may have the same function as BdBAHD01, as suppression and overexpression of this gene induced respective decreases and increases in FA (Buanafina *et al*., 2016). Of total BAHD01 and BAHD05 transcript abundance in stems of control plants, SvBAHD01 accounts for 83% in *Setaria* whereas BdBAHD01 only accounts for 56% in *Brachypodium*. There is no evidence of a compensatory upregulation of other BAHD transcripts in response to BAHD01 suppression in either species (Fig. 3). The relative lack of effect of BdBAHD01 suppression may therefore be due to greater redundancy in *Brachypodium*, or simply that the suppression was insufficient to limit feruloylation in this species; identification of BdBAHD01 knock‐out mutants would address this possibility.

Other genes within the phenylpropanoid pathway leading to monolignol biosynthesis were not differentially expressed in SvBAHD01 RNAi stems (Notes S1) and total lignin was unaffected in stems (Fig. 2d). We found evidence from 2D‐NMR of decreased **S**/**G** ratio in stems but not leaves (Fig. 5); estimates of **H** lignin units are not reliable from this method due to overlapping protein signals (Kim *et al*., 2017). We assumed, as is common, that all FA and diFA released by mild alkali from AIR is ester‐linked to arabinofuranose on GAX. In fact, as monolignol ferulates are now firmly established monomers in the lignification of monocots, such compounds could in principle also result from this lignin source; however, as ferulates and diferulates are well incorporated into lignins by radical coupling reactions, the extremely low released concentrations of such components can be neglected here (Karlen *et al*., 2016). This assumption was supported by quantitation of Ara*f*‐FA released by mild acidolysis from SvBAHD01 RNAi lines (Fig. 4). We observed similar relative effects of SvBAHD01 silencing as for total FA, decreases of 65–65% from stems and 30–35% from leaves in T_3_ (Fig. 4B) and T_4_ (Fig. S3) generation plants. An unexpected result was the increase in *p*CA observed in leaves, but not stems, of both SvBAHD01 RNAi lines (Fig. 2b). There are two forms of ester‐linked *p*CA in grass cell walls: those acylating Ara*f* on GAX and those acylating the lignin sidechain (Ralph, 2010). Ara*f*‐*p*CA content more than doubled due to SvBAHD01 suppression in both leaves and stems (Fig. 4b), but this only accounts for a proportion of the total *p*CA in leaves, and a smaller proportion in stems. BAHD genes such as OsAT10 (our BAHD10) are probably responsible for the Ara*f*‐*p*CA whereas the BAHD PMT genes are responsible for the addition of *p*CA to monolignols, and hence its appearance on lignin, but none of the BAHD genes showed significant upregulation in SvBAHD01 RNAi plants (Fig. 3). One possible explanation for the increased cell wall *p*CA is that the blocking of addition of FA to Ara*f* results in a build‐up of FA‐CoA and *p*CA‐CoA substrates; the increased *p*CA‐CoA concentration results in more *p*CA addition to GAX in both stems and leaves. There also seems to be increased lignin‐*p*CA in leaves of SvBAHD01 RNAi plants; in stems, SvBAHD09 transcript, a putative PMT gene, was downregulated (Fig. 3), which could be a regulatory response to prevent excessive addition of *p*CA to lignin.

All BAHD proteins are believed to be localized to the cytosol and this has been confirmed using a green fluorescent protein (GFP) fusion of TaBAHD01 for the wheat ortholog (J. Freeman, unpublished), but feruloylation of GAX takes place within the Golgi (Myton & Fry, 1994; Fry, 2004). It has therefore been suggested (Buanafina, 2009) that a cytosolic precursor such as UDP‐Ara*f* is the acceptor for FA or *p*CA, as their CoA thioesters, mediated by these BAHD enzymes; as UDP‐Ara*f* is generated in the cytosol by UDP‐arabinopyranose mutase, this is feasible. The feruloylated UDP‐Ara*f* would then pass into the Golgi by a transporter (possibly encoded by grass homologs of the recently identified UDP‐Ara*f* transporters in Arabidopsis; Rautengarten *et al*., 2017) and FA‐Ara*f* would be transferred onto GAX, probably by GT61 enzymes that are responsible for arabinosylation of xylan (Anders *et al*., 2012). In support of this model, RNAi *Brachypodium* lines with decreased mutase (Rancour *et al*., 2015) and the rice *xax1* mutant which carries a knockout for a GT61 family gene (Chiniquy *et al*., 2012) both showed substantial decreases in cell wall FA; in contrast to our results for SvBAHD01 suppression, they also both showed decreased cell‐wall *p*CA. This may suggest that specificity for FA or *p*CA is conferred exclusively by the BAHD enzymes in this pathway.

Biomass production was unaffected by SvBAHD01 suppression (Fig. 6a) and ease of saccharification was increased (Fig. 6d). However, there were some pleiotropic effects; the many differentially regulated transcripts in the SvBAHD01 RNAi lines suggest shifts in development, protein synthesis and increased stress responses (Notes S1) and there was significantly decreased seed size (Fig. 6b) and changes in stem morphology (Fig. 6e). Ferulate‐mediated cross‐linking is fundamental to both primary and secondary cell walls in grasses and it is not surprising that constitutive suppression has downstream consequences; directing the suppression to secondary cell walls specifically (as achieved elsewhere; Yang *et al*., 2013) might decrease pleiotropic effects whilst maintaining a benefit in digestibility. The large increase in biomass saccharification that we observed in SvBAHD01 RNAi plants (Fig. 6d) indicates that BAHD01 represents a promising target to increase the suitability of grass biomass for biofuel and animal feed applications. The effect appears relatively specific to affecting FA linked to GAX but not total lignin (Fig. 2d), compared with modification of genes responsible for earlier steps in the phenylpropanoid pathway (Bouvier d'Yvoire *et al*., 2013). We would predict that it results in fewer covalent linkages between the polysaccharide and lignin components of cell walls (as well as between polysaccharides themselves), allowing greater ease of separation, for example, for biorefining, but this remains to be demonstrated. Indeed, it has been demonstrated that, in a model system, the rate and extent of wall hydrolysis by fungal enzymes is affected by ferulate‐mediated polysaccharide cross‐linking (Grabber *et al*., 1998a) and even more by lignin‐polysaccharide cross‐linking (reviewed by Ralph *et al*., 1998; Ralph, 2010). Greater understanding of the role of BAHD01 and related genes will help to identify opportunities for grass crop improvement and elucidate the importance of cell‐wall feruloylation in grass evolution.

## Author contributions

H.B.C.M., J.F., R.A.C.M. and T.K.P. planned and designed the research. W.R.d.S., P.K.M., J.F., T.K.P., L.V.M., B.L.S., F.V., A.P.R., B.A.D.B.d.C., A.K.K., P.A.d.O., R.B.C., T.F.P., D.C.I.M., R.M., O.F‐F., W.D.d.S., R.T., F.M.S., D.d.C., M.G., M.R.B., M.A.S.T., J.R., R.A.C.M. and H.B.C.M. performed research and/or analyzed the data. R.A.C.M. wrote the manuscript with contributions from H.B.C.M., J.R., P.K.M. and W.R.d.S.

## Supporting information

Please note: Wiley Blackwell are not responsible for the content or functionality of any Supporting Information supplied by the authors. Any queries (other than missing material) should be directed to the *New Phytologist* Central Office.

**Fig. S1** RNAi construct, and HCA content of *Brachypodium* and *Setaria* RNAi plants.
**Fig. S2** LC‐MS chromatograms and correlations of UV absorbance and MRM ion count peak areas.
**Fig. S3** HCA‐Ara content for T_4_ *Setaria* samples.
**Table S1** SvBAHD01 silencing, FA content and segregation in *Setaria* SvBAHD01 RNAi lines
**Table S2** HCA content of *Brachypodium* and *Setaria* samples
**Table S3** MRMs and identities of most prevalent ions released from AIR samples by mild acidolysis
**Table S4** HCA content of saponified samples following mild acidolysis from *Setaria* plants
**Table S5** Monosaccharide composition of cell walls from *Setaria* plants
**Methods S1** Procedures for plant growth, microscopy and determination of gene expression, cell‐wall monosaccharides, lignin and biomass.

**Notes S1** Differentially expressed transcripts in SvBAHD01 RNAi stems from RNA‐seq.
