# Supplementary material for: Suppression of a single BAHD gene in Setaria viridis causes large, stable decreases in cell wall feruloylation and increases biomass digestibility
Source: New Phytol. 2018 Jan 8;218(1):81–93. doi: 10.1111/nph.14970 (PMC5873385; doi:10.1111/nph.14970)
Supplement: Supplementary file 1 — Fig. S1 RNAi construct, and HCA content of Brachypodium and Setaria RNAi plants. Fig. S2 LC‐MS chromatograms and correlations of UV absorbance and MRM ion count peak areas. Fig. S3 HCA‐Ara content for T4 Setaria samples. Table S1 SvBAHD01 silencing, FA content and segregation in Setaria SvBAHD01 RNAi lines Table S2 HCA content of Brachypodium and Setaria samples Table S3 MRMs and identities of most prevalent ions released from AIR samples by mild acidolysis Table S4 HCA content of saponified samples following mild acidolysis from Setaria plants Table S5 Monosaccharide composition of cell walls from Setaria plants Methods S1 Procedures for plant growth, microscopy and determination of gene expression, cell‐wall monosaccharides, lignin and biomass. [file NPH-218-81-s001.pdf]

Suppression of a single BAHD gene in *Setaria viridis* causes large, stable decreases in cell wall feruloylation and increases biomass digestibility

Wagner R. de Souza, Polyana K. Martins, Jackie Freeman, Till K. Pellny, Louise V. Michaelson, Bruno L. Sampaio, Felipe Vinecky, Ana P. Ribeiro, Barbara A. D. B. da Cunha, Adilson K. Kobayashi, Patricia A. de Oliveira, Raquel B. Campanha, Thályta F. Pacheco, Danielly C.I. Martarello, Rogério Marchiosi, Osvaldo Ferrarese-Filho, Wanderley D. dos Santos, Robson Tramontina , Fabio M. Squina, Danilo C. Centeno, Marília Gaspar, Marcia R. Braga, Marco A. S. Tiné, John Ralph, Rowan A. C. Mitchell, Hugo B. C. Molinari

Article accepted: 22 November 2017

SUPPORTING INFORMATION

Methods S1

Growth conditions for plants

Brachypodium plants were grown under 14/10h day (25/20 °C) after three weeks vernalisation at 4 °C. Setaria plants were maintained under 16 h photoperiod of 400 μmol $m^{-2}s^{-1}$ light intensity, 26±2 °C and 65% relative humidity until harvested at the reproductive phase. All analyses were performed in this phase, except biomass and seed data which was collected at maturity.

Gene expression analysis by Real-Time qPCR

Leaves of the thirteen events (T_0) and the leaf and stem tissues of T_3 events 17.3, 18.1 and NT were harvested at the reproductive phase. Pools of three T_3 plants for each tissue were harvested, comprising three biological samples. Isolation of total RNA from tissues, cDNA synthesis and RT-qPCR using SYBR green was performed as described by (Martins *et al.*, 2016). The primers used in the RT-qPCR for *SvBAHD01* amplification were SvBAHD01-F 5´GGGTTCTCGGAGGTGGACTA 3´ and SvBAHD01-R 5´TGACGACAGAGGCGATGAAG 3´.

Quantification of cell-wall-bound hydroxycinnamate content

Protocol in Rothamsted lab (Fig. 2, Table S2, Fig. S1D): Cell-wall-bound phenolics were extracted from 10 mg freeze-dried ground tissue as previously described (Pellny *et al.*, 2012) except that the AIR preparation included two extractions with

chloroform:methanol; 3:2 after ethanol extractions. Samples were dissolved in 500 µL 50% methanol:2% acetic acid and quantified by HPLC on a Shimadzu Prominence high-performance liquid chromatograph with a photo-diode array detector using a UPLC Kinetex Phenyl-Hexyl (150 x 4.6 mm, 5 µm) column as described (Freeman *et al.*, 2017). Relative response factors for diferulates 8-8´ aryltetralin form (8-8´ AT), 8-8' plus 8-8´ tetrahydrofuran form (8-8´ THF), 8-5´, 8-5´ benzofuran form (8-5' BF), 5-5´ and 8-O-4´ were 0.327, 0.429, 0.456, 0.501, 0.417 and 0.692 respectively.

Protocol in Embrapa Agroenergy lab (Table S1, Fig. S1C): Aliquots of 20.0 ± 0.2 mg (in triplicate) of freeze-dried ground tissues (leaf and stem) of each biological replicate were extracted with 80% ethanol (v/v) in an ultrasonic bath at 40 ºC for 10 min, centrifuged at 1,900 x *g* for 5 min and the supernatant was transferred to other microcentrifuge tube. This procedure was repeated twice to obtainment of AIR of each sample. To the AIRs was added 20 µL of an internal standard (3,5-dichloro-4-hydroxybenzoic acid, 1.5 mg.mL^{-1}) prior the alkaline hydrolysis with 2 M NaOH solution (800 µL) in nitrogen atmosphere for 18 h in the dark. After the alkaline hydrolysis, samples were centrifuged at 5,000 x *g* for 15 min, an aliquot of 600 µL of the supernatants were transferred to 2 mL microcentrifuge tubes, acidified with 12 M HCl (220 µL) to pH 2.0, and extracted with ethyl acetate (800 µL x 3). The ethyl acetate extracts were dissolved in 1 mL of a mixture of ultrapure water (Milli-Q)/acetonitrile (HPLC grade) (7:3, v/v), in vortex (30 s), followed by ultrasonic bath for 5 min, then centrifuged at 1,900 x *g* for 5 min. The supernatant (800 µL) was filtered through a syringe filter (0.22 µm) into HPLC vials to be analyzed by UHPLC-DAD. Chromatographic analysis of the samples was performed on a Waters® ACQUITY UPLC HSS T3 reverse phase analytical column (150 mm L. x 2.1 mm i.d., 1.8 µm particle size) placed in an oven maintained at 40 °C using H_2O with 0.05% trifluoroacetic acid as solvent A and acetonitrile as solvent B. The following gradient elution program was used: at 0 min, it started with 15% B which was increased to 30% B in 5 min, then to 100% B in 7 min, continued at 100% B to 9 min for washing and was equilibrated back to 15% B from 9.01 to 13 min, at a solvent flow rate of 600 µL/min. The phenolic acids were identified in an Agilent® Model 1290 Diode Array Detector. Quantitation of phenolic acids was by integration of peak areas at 280 nm with reference to calibrations made using known amounts of pure compounds, using the area ratio of the phenolic acids peaks to the I.S. peak.

Lignin quantification

Dry samples (0.3 g) were homogenized in 50 mM potassium phosphate buffer (7 mL, pH 7.0) and transferred into a centrifuge tube. The pellet was centrifuged (1,400 x g, 4 min) and washed by successive stirring and centrifugation as follows: eight times with phosphate buffer (pH 7.0; 7 mL), four times with 1% (v/v) Triton X-100 in pH 7.0 buffer (7 mL), six times with 1 M NaCl in pH 7.0 buffer (7 mL), three times with distilled water (7 mL), and two times with acetone (5 mL). The pellet was dried in an oven (60 °C, 24 h) and cooled in a vacuum desiccator. The dry matter obtained was defined as the protein-free cell wall fraction. Protein-free cell wall sample (20 mg) was placed into a screw-cap centrifuge tube containing 0.5 mL of 25% acetyl bromide (v/v in glacial acetic acid) and incubated at 70 °C for 30 min. After complete digestion, the sample was quickly cooled in an ice bath, and then mixed with 0.9 mL of 2 M NaOH, 0.1 mL of 5 M hydroxylamine-HCl, and a volume of glacial acetic acid sufficient for complete solubilization of the lignin extract (4 mL for leaves and stems). After centrifugation (1,400 x g, 4 min), the absorbance of the supernatant was measured at 280 nm. A standard curve was generated with alkali lignin (Aldrich 37, 096-7) and the absorptivity (ε) value obtained was 22.9 g^{-1} L cm^{-1}. The results were expressed as mg lignin g^{-1} cell wall.

Cell wall characterization by solution-state 2D NMR

Setaria cell walls were characterized using solution-state 2D NMR according to procedure described by (Kim & Ralph, 2010). Aliquots of approximately 500 mg (in triplicate) of freeze-dried ground tissues (leaf, stem and root) of each biological replicate from NT control and events 17.3 and 18.1 were weighed and extracted overnight (minimum of 8 h) with a mixture acetone/water (95:5 v/v) on a Soxhlet apparatus (~70 ºC). The extract-free samples were oven-dried at 50 ºC for 48 h. The dried extract-free plant material was submitted to a ball-milling procedure. Aliquots of 200 mg of each sample were milled in 20 mL jars with 10 x 10 mm ball bearings in a Fritsch® Planetary micro mill Pulverisette 7 premium line equipment, according to following milling protocol: 5 x 5 min with 5 min pauses in between. After the ball-milling procedure, the preparation for NMR analysis for all samples was carried out according to (Kim & Ralph, 2010) for gelling samples without derivatization, and their conditions

used for acquisition of the NMR spectra and processing. The acquisition of the NMR spectra were performed at Laboratory of Nuclear Magnetic Resonance of Federal University of São Carlos (São Carlos-SP/Brazil), on a 600 MHz Bruker® AVANCE III spectrometer system equipped with a 5 mm TCI cryoprobe with ATMA® (Automatic Tuning and MAtching).

Biomass measurement

For biomass sampling, 10 plants were selected for each event (Ev 17.3 and Ev 18.1) and for NT. The plant organs (excluding root) were completely dried in an oven at 65 °C for about 72 h to obtain the aboveground dry biomass at maturity. The seeds were collected after 90 days and the weight of one thousand seeds was estimated.

Stem sectioning and Microscopy

Stem samples from 3 replicate plants per line were fixed in 50% FAA and stored in 70% ethanol. After tissue dehydration in ethanol, the samples were prepared with HistoResin (Leica) according to manufacturer's instructions. Transversal sections of 10 µm were obtained using a rotation microtome. The sections were treated with auramine O (0.01% in distilled water) for 20 s, according to (Considine & Knox, 1979) and Caucofluor white (0.01% in distilled water) for 5 min. The cross-sections were capped an analyzed in an Axioskop 2 plus fluorescence microscope under 365 nm UV light. For Wiesner test, cross-sections were incubated for 10 min in phloroglucinol-HCl and immediately photographed with a digital camera coupled to a Leica ICC50 light microscope, using Leica Application Suite version 1.8.

Table S1. Setaria SvBAHD01 transgenic lines. qRT-PCR of SvBAHD01 leaves and their related ferulic acid (FA) reduction at T_0 generation and segregation of T_1 generation. Segregation analysis was performed according to Martins et al. (2015).

Event (T_0)	Silencing (%)	FA reduction (%)	Event (T_1)[a]	Resistant[b]	Sensitive[c]	X^2 value for 3:1[d]	Fits 3:1[e]
Ev. 4	61.55	55.57	-	-	-	-	-
Ev. 8	61.08	38.51	8.9	83	17	3.41	Y
Ev. 10	56.15	56.01	-	-	-	-	-
Ev. 17	96.12	56.23	17.3	72	32	1.84	Y
Ev. 18	99.32	57.76	18.1	62	28	1.79	Y
Ev. 19	80.43	52.73	-	-	-	-	-
Ev. 21	88.36	60.39	21.8	30	8	0.31	Y

[a] Transgenic events of plants at T_1 generation chosen for segregation analysis based on silencing and ferulic acid (FA) levels.
[b] Number of seedlings survived on the medium containing 50 mg/L hygromycin.
[c] Number of seedlings sensitive on the medium containing 50 mg/L hygromycin.
[d] Chi-square (X^2) statistical test for segregation analysis. Values represent statistical significance at $p < 0.05$.
[e] Ratio of resistant versus sensitive seedlings on hygromycin.
Y: yes; N: no.

Table S2. HCAs in Brachypodium leaves and stems of T_2 plants descended from six transformation events that are azygous (A) or homozygous (H) for BdBAHD01 RNAi transgene. HCA content is expressed per unit dry wt of tissue. Paired t-test was used to assess effect of transgene. Lines B2 and B5 were selected for further analysis.

		leaves				stems			
line name	Trans-gene	µg *p*CA/ mg	µg FA/ mg	diF/IS/ mg	FA/*p*CA	µg *p*CA/ mg	µg FA/ mg	diF/IS/ mg	FA/*p*CA
B2	A	1.14	3.25	0.09	2.92	3.75	4.65	0.09	1.26
B3	A	1.25	3.67	0.10	3.00	3.44	4.64	0.09	1.38
B5	A	1.38	3.83	0.10	2.85	3.23	4.05	0.08	1.28
B13	A	1.26	3.82	0.10	3.11	3.94	5.16	0.11	1.34
B15	A	1.26	3.58	0.11	2.93	4.03	5.16	0.11	1.31
B16	A	1.37	4.07	0.10	3.06	2.83	3.67	0.07	1.32
B2	H	1.26	3.09	0.10	2.52	4.06	4.45	0.10	1.12
B3	H	1.49	3.34	0.09	2.30	4.79	4.63	0.10	0.99
B5	H	1.31	3.24	0.10	2.55	2.63	2.72	0.06	1.06
B13	H	1.22	3.58	0.10	3.01	4.19	5.30	0.11	1.29
B15	H	1.30	3.74	0.10	2.96	2.95	3.75	0.07	1.30
B16	H	1.27	3.80	0.09	3.06	4.29	4.91	0.10	1.17
Paired ttest P-value		0.55	0.06	0.04	0.08	0.53	0.55	0.62	0.03
av. H/A %		103%	94%	97%	92%	110%	95%	96%	88%

Table S3. MRMs and identities of most prevalent ions released from AIR samples by mild acidolysis. Fragmentation of parent ion m/z 325.09 was essentially identical to that of pure Ara*f*-FA standard (Quemener & Ralet, 2004) confirming its identity. Parent ion m/z 295.1 had the same fragmentation pattern with masses 30.0 less for fragments containing HCA, confirming its identity as Ara*f*-*p*CA. FA and *p*CA confirmed from pure standards.

Q1 Mass	***Q3 Mass***	***Time mS***	***ID***	***DP***	***EP***	***CE***	***CXP***
325.09	265.1	20	Ara-FA (-$C_2H_4O_2$)	-50	-2	-20	-1
325.09	193.05	20	Ara-FA (-$C_5H_8O_4$)	-50	-2	-20	-1
325.09	183.05	20	Ara-FA (-$C_7H_{10}O_3$)	-50	-2	-20	-1
295.1	235.1	20	Ara-*p*CA (-$C_2H_4O_2$)	-22	-5	-20	-2
295.1	163.04	20	Ara-*p*CA (-$C_5H_8O_4$)	-22	-5	-20	-2
295.1	153.04	20	Ara-*p*CA (-$C_7H_{10}O_3$)	-22	-5	-20	-2
193	149	20	FA	-50	-2	-20	-1
163	118.9	20	*p*CA	-22	-5	-20	-2
457.1	265.1	20	Xyl-Ara-FA (-Xyl- $C_2H_4O_2$)	-100	-10	-50	-5
457.1	193	20	Xyl-Ara-FA (-Xyl- $C_5H_8O_4$)	-100	-10	-50	-5
457.1	439.1	20	Xyl-Ara-FA (-$C_2H_4O_2$)	-100	-10	-50	-5
427.1	163	20	Xyl-Ara-*p*CA (-Xyl- $C_5H_8O_4$)	-100	-10	-50	-5
649.18	589.18	20	Ara-diFA-Ara (-$C_2H_4O_2$)	-100	-10	-50	-5
439	113	20	*TFA-Ara-FA (-Ara FA +H2O)	-50	-2	-20	-1
409	113	20	*TFA-Ara-*p*CA (-Ara *p*CA +H_2O)	-22	-5	-20	-2

*Putative trifluoroacetic acid (TFA) adducts of major ions.

Table S4. HCA content of saponified samples following mild acidolysis from T_3 and T_4 generation Setaria transgenic and control plants. Values are means ± SEM, n=3. Ratios of transgenics relative to control are given with P-value of corresponding t-test indicated (* P<0.05, ** P<0.001). † Proportion of total HCA (supernatant + pellet) present as HCA-Ara conjugate from values shown in Fig. 4. *p*CA and FA are given as µg (mg AIR)$^{-1}$ for T_3 and rel. peak area (mg AIR)$^{-1}$ for T_4 samples.

		*p*CA µg (mg AIR)$^{-1}$			
T_3 generation	NT	Ev17.3	Ev18.1	Ev17.3 / NT	Ev18.1 / NT
leaves supernatant	2.92 ± 0.06	6.73 ± 0.27	5.41 ± 0.03	231%**	185%**
leaves pellet	1.51 ± 0.04	3.56 ± 0.09	3.70 ± 0.06	235%*	245%**
stems supernatant	2.67 ± 0.48	2.45 ± 0.04	2.27 ± 0.16	92%*	85%*
stems pellet	7.95 ± 0.92	9.79 ± 0.19	8.95 ± 0.09	123%**	113%**

		*p*CA-Ara / *p*CA†			
T_3 generation	NT	Ev17.3	Ev18.1	Ev17.3 / NT	Ev18.1 / NT
leaves	34% ± 0%	39% ± 1%	34% ± 1%	114%*	98%
stems	3% ± 0%	5% ± 0%	6% ± 0%	185%**	242%**

		FA µg (mg AIR)$^{-1}$			
T_3 generation	NT	Ev17.3	Ev18.1	Ev17.3 / NT	Ev18.1 / NT
leaves supernatant	8.22 ± 0.15	5.96 ± 0.19	5.26 ± 0.13	72%**	64%**
leaves pellet	0.72 ± 0.02	0.56 ± 0.01	0.52 ± 0.01	78%**	71%**
stems supernatant	7.94 ± 0.69	2.61 ± 0.08	2.18 ± 0.19	33%**	27%**
stems pellet	1.17 ± 0.08	0.49 ± 0.01	0.36 ± 0.01	42%**	31%**

		FA-Ara / FA†			
T_3 generation	NT	Ev17.3	Ev18.1	Ev17.3 / NT	Ev18.1 / NT
leaves	40% ± 1%	40% ± 2%	40% ± 1%	100%	99%
stems	37% ± 2%	35% ± 1%	39% ± 2%	95%*	105%

		*p*CA rel. peak area (mg AIR)$^{-1}$			
T_4 generation	NT	Ev17.3	Ev18.1	Ev17.3 / NT	Ev18.1 / NT
leaves supernatant	0.123 ± 0.005	0.170 ± 0.012	0.140 ± 0.007	137%*	114%*
leaves pellet	0.243 ± 0.014	0.317 ± 0.011	0.302 ± 0.010	130%*	124%*
stems supernatant	0.101 ± 0.003	0.101 ± 0.003	0.105 ± 0.015	92%	95%
stems pellet	0.806 ±0.070	0.906 ± 0.034	0.786 ± 0.018	112%*	98%

		FA rel. peak area (mg AIR)$^{-1}$			
T_4 generation	NT	Ev17.3	Ev18.1	Ev17.3 / NT	Ev18.1 / NT
leaves supernatant	0.180 ± 0.009	0.122 ± 0.008	0.121 ± 0.005	67%**	67%**
leaves pellet	0.071 ± 0.004	0.050 ± 0.002	0.057 ± 0.002	70%**	81%**
stems supernatant	0.189 ± 0.004	0.078 ± 0.002	0.083 ± 0.011	41%**	44%**
stems pellet	0.081 ± 0.006	0.035 ± 0.001	0.039 ± 0.002	44%**	48%**

Table S5. Monosaccharide and acetyl composition of AIR from leaves (L- samples) and stems (S- samples) of control and *SvBAHD01* RNAi transgenic plants descended from 17.3 and 18.1 events. Values are means ± SEM with significance of difference of transgenic from control indicated if difference in means > LSD from ANOVA at *P<0.05, **P<0.01, ***P<0.001).

Sample	% AIR														
	Arabinose			Galactose			Glucose			Xylose			Acetyl		
	Main		SE	Main		SE	Main		SE	Main		SE	Main		SE
L-NT	3.99	±	0.06	1.21	±	0.03	32.70	±	0.34	13.59	±	0.06	1.99	±	0.07
L-17.3	4.53	±	0.04*****	1.39	±	0.02*****	34.94	±	0.41	14.49	±	0.15*****	2.16	±	0.03*****
L-18.1	3.99	±	0.04	1.25	±	0.01	33.09	±	0.08	13.37	±	0.10	2.03	±	0.04
S-NT	4.49	±	0.06	1.39	±	0.05	35.75	±	0.33	20.14	±	0.06	5.14	±	0.03
S-17.3	4.53	±	0.05	1.52	±	0.06	35.83	±	0.13	19.07	±	0.15	5.33	±	0.08
S-18.1	4.41	±	0.05	1.68	±	0.03*****	33.24	±	0.35	18.20	±	0.25*****	4.48	±	0.06*****

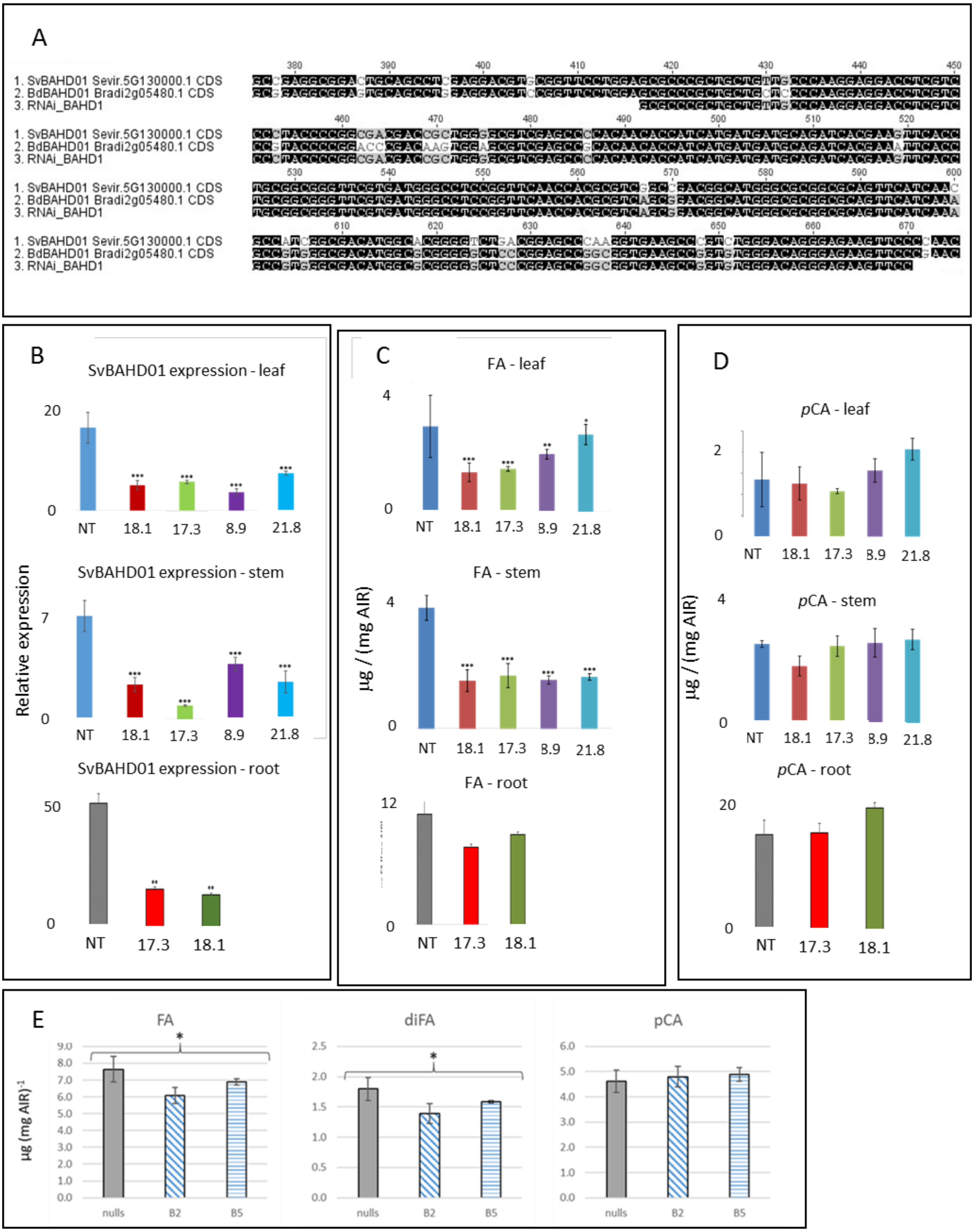

Figure S1. A: Alignment of region targeted by BAHD01 RNAi (RNAi_BAHD1) and

Setaria and Brachypodium BAHD01 genes. RNAi_BAHD1 matches 100% to SvBAHD01 CDS sequence 333-482 and 100% to BdBAHD01 CDS sequence 520-670. SvBAHD01 expression (B) and relative amounts of FA (C) and *p*CA (D) from Setaria events at T_2 generation in different tissues. B-D: significance of difference of transgenic from control indicated if difference in means > LSD from ANOVA at *P<0.05, **P<0.01, ***P<0.001. E: HCA content of AIR isolated from stems of two homozygous transgenic lines of Brachypodium expressing BAHD1 RNAi ("B2", "B5") and azygous null segregants ("nulls"). Error bars are 95% confidence intervals calculated for nulls (n=13) and B2 and B5 lines (n=3) each. FA monomer and diFA content was significantly decreased in transgenics compared to nulls (F test; comparing all nulls to all transgenics; P<0.05, indicated by *). *p*CA content was not significantly altered.

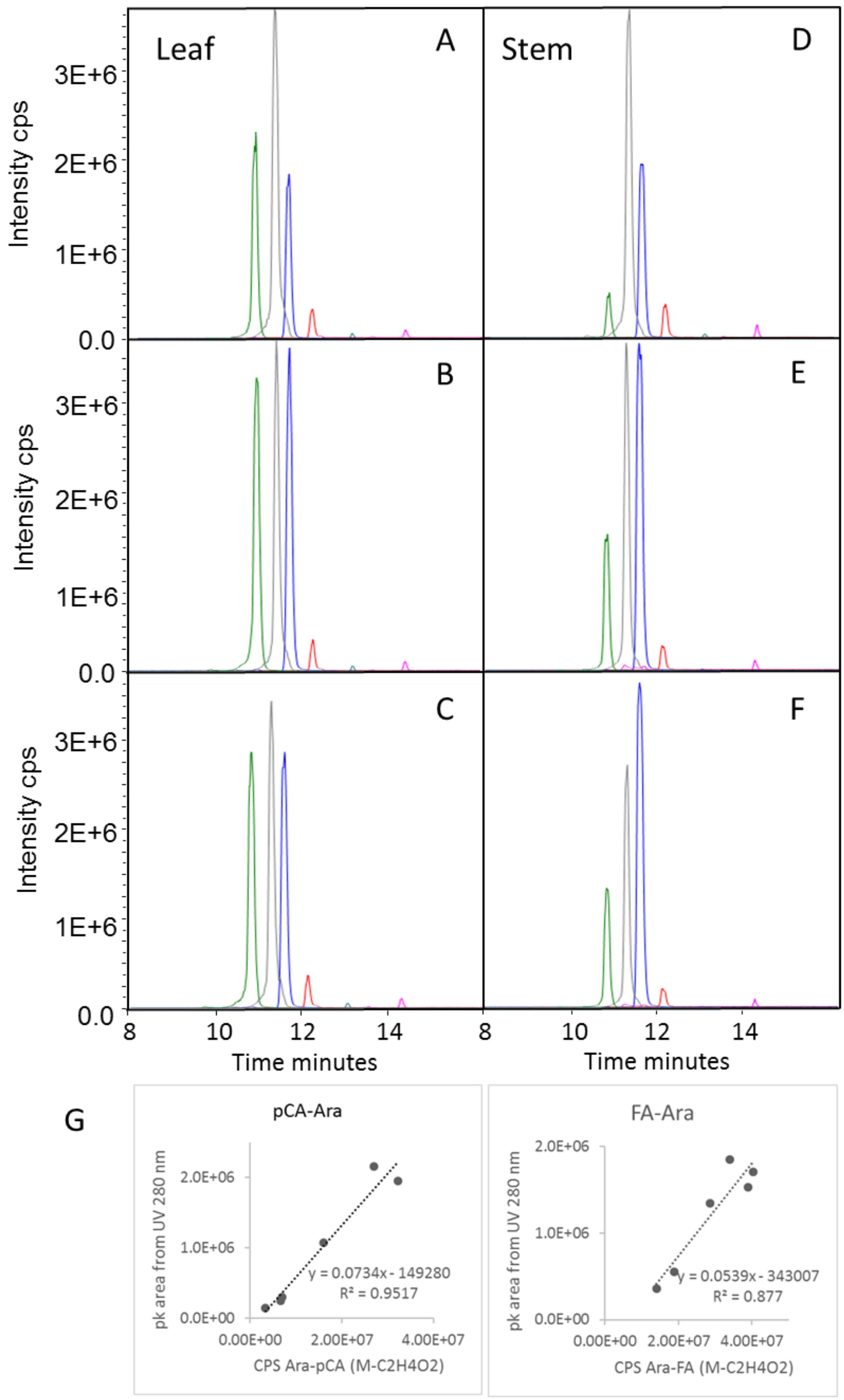

Figure S2. Panels A-F: MRM Chromatogram of HCA-conjugates following mild acidolysis of Sertaria AIR from NT (A & D), 17.3 (B & E), 18.1 plants (C & F). Colours as follows Ara-pCA (-$C_2H_4O_2$)– green and Ara-FA (-$C_2H_4O_2$)- grey, pCA- blue, FA- red, Ara-diFA-Ara (-$C_2H_4O_2$) – emerald, Xyl-Ara-FA (-Xyl- $C_5H_8O_4$) – pink. MRM parent and fragment ions are shown in Table S3. All peaks were subject to a Gaussian smooth,

with the distance between points set to a minimum of 10. Panel G: Correlation of MRM peak areas with UV peak areas of Ara-pCA and Ara-FA for same samples.

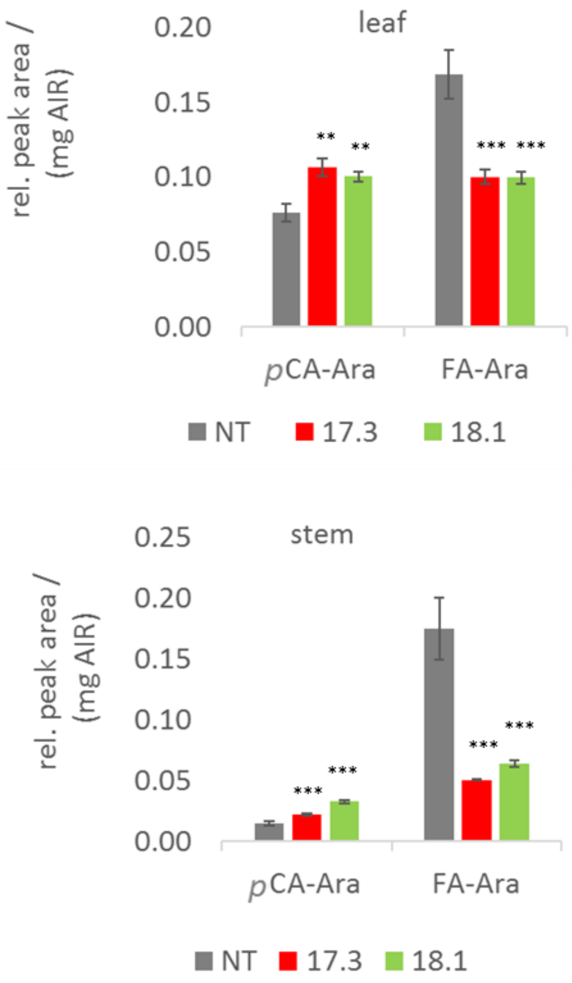

Figure S3. HCA-Ara content for T_4 and control Setaria plant samples using same methodology as in Fig. 4. Mean *p*CA-Ara and FA-Ara contents expressed as peak area (absorbance at 280 nm) relative to that of internal standard per mg AIR (n=4; error bars SEM, significance of difference of transgenic from control indicated if difference in means > LSD from ANOVA at *P<0.05, **P<0.01, ***P<0.001).

References

Freeman J, Ward JL, Kosik O, Lovegrove A, Wilkinson MD, Shewry PR, Mitchell RAC. 2017. Feruloylation and structure of arabinoxylan in wheat endosperm cell walls from RNAi lines with suppression of genes responsible for backbone synthesis and decoration. *Plant Biotechnol J* **15**(11): 1429–1438.

Kim H, Ralph J. 2010. Solution-state 2D NMR of ball-milled plant cell wall gels in DMSO-d_6/pyridine-d_5. *Organic & Biomolecular Chemistry* **8**(3): 576-591.

Martins PK, Mafra V, de Souza WR, Ribeiro AP, Vinecky F, Basso MF, da Cunha BADB, Kobayashi AK, Molinari HBC. 2016. Selection of reliable reference genes for RT-qPCR analysis during developmental stages and abiotic stress in Setaria viridis. *Scientific Reports* **6**: 28348

Martins PK, Ribeiro AP, Cunha BADBd, Kobayashi AK, Molinari HBC. 2015. A simple and highly efficient Agrobacterium-mediated transformation protocol for Setaria viridis. *Biotechnology Reports* **6**: 41-44.

Pellny TK, Lovegrove A, Freeman J, Tosi P, Love CG, Knox JP, Shewry PR, Mitchell RAC. 2012. Cell walls of developing wheat starchy endosperm: comparison of composition and RNA-seq transcriptome. *Plant Physiology* **158**(2): 612-627.
